# Task-Shifting in HIV Care: A Case Study of Nurse-Centered Community-Based Care in Rural Haiti

**DOI:** 10.1371/journal.pone.0019276

**Published:** 2011-05-06

**Authors:** Louise C. Ivers, Jean-Gregory Jerome, Kimberly A. Cullen, Wesler Lambert, Francesca Celletti, Badara Samb

**Affiliations:** 1 Brigham and Women's Hospital, Boston, Massachusetts, United States of America; 2 Harvard Medical School, Boston, Massachusetts, United States of America; 3 Partners In Health, Boston, Massachusetts, United States of America; 4 Harvard Medical School Center for AIDS Research, Boston, Massachusetts, United States of America; 5 Zanmi Lasante, Cange, Haiti; 6 World Health Organization, Geneva, Switzerland; Tulane University, United States of America

## Abstract

**Introduction:**

At least 36 countries are suffering from severe shortages of healthcare workers and this crisis of human resources in developing countries is a major obstacle to scale-up of HIV care. We performed a case study to evaluate a health service delivery model where a task-shifting approach to HIV care had been undertaken with tasks shifted from doctors to nurses and community health workers in rural Haiti.

**Methods:**

Data were collected using mixed quantitative and qualitative methods at three clinics in rural Haiti. Distribution of tasks for HIV services delivery; types of tasks performed by different cadres of healthcare workers; HIV program outcomes; access to HIV care and acceptability of the model to staff were measured.

**Results:**

A shift of tasks occurred from doctors to nurses and to community health workers compared to a traditional doctor-based model of care. Nurses performed most HIV-related tasks except initiation of TB therapy for smear-negative suspects with HIV. Community health workers were involved in over half of HIV-related tasks. HIV services were rapidly scaled-up in the areas served; loss to follow-up of patients living with HIV was less than 5% at 24 months and staff were satisfied with the model of care.

**Conclusion:**

Task-shifting using a community-based, nurse-centered model of HIV care in rural Haiti is an effective model for scale-up of HIV services with good clinical and program outcomes. Community health workers can provide essential health services that are otherwise unavailable particularly in rural, poor areas.

## Introduction

The healthcare worker crisis in developing countries is a major obstacle to scale-up of HIV care [1]–[4]. In 2006, the World Health Organization (WHO) reported that 36 countries in sub-Saharan Africa suffer from a severe shortage of health workers such as doctors, nurses, pharmacists, and other support staff. Other, non-African countries are also suffering from similar serious shortages [5], [6]. For HIV and non-HIV health services to be effectively scaled up globally, appropriate strategies to address this human resource crisis must be adopted. One strategy of interest is ‘task-shifting’: a rational re-distribution of tasks among healthcare teams—including the delegation of tasks, where appropriate, to health workers with less training than those traditionally assigned to that task.

In August 2006, WHO launched a coordinated global effort called “Treat, Train and Retain” to address this major barrier to HIV care and prevention [7]. In concert with this, WHO commissioned a study to document the reorganization of service delivery in several countries, specifically to document the practice of task-shifting and its impact on HIV services delivery. This report describes the results of a study undertaken in Haiti.

Partners In Health is a non-governmental organization with a mission to provide healthcare to the poor. Zanmi Lasante, Partners In Health's sister organization in Haiti has been involved in the care of patients with HIV infection in central Haiti since the early days of the epidemic; programs are collaborative with the ministry of health, care is provided free of charge and community-based care is the mainstay of the model. The model has been described in detail previously [8], [9].

The study objective was to determine the extent to which tasks traditionally performed by doctors were shifted to or shared with nurses and the extent to which tasks traditionally for nurses were shifted and shared with other cadres. Secondary objectives were to evaluate the acceptability of the model to staff and to report on the outcomes of the clinical program using this model of HIV care.

## Methods

### Ethics Statement

The study protocol was reviewed and approved by the Institutional Review Boards of the Brigham and Women's Hospital and the Zanmi Lasante Ethics Committee.

We used mixed qualitative and quantitative methods to perform a cross-sectional observational case study at three sites in Haiti. A list of tasks representing those typically performed in HIV care was developed by the WHO. It was field-tested and adapted during focus groups in Haiti to ensure that a broad array of outpatient HIV-related services and activities were represented. In order to compare findings of this study to an historical or more ‘traditional’ doctor-based model of HIV care, HIV physicians practicing in resource-limited settings were polled to determine the distribution of tasks that was most consistent with ‘traditional’ HIV care before task-shifting had been instituted. A summary of their responses provided a baseline to which we compared the model of HIV care being studied. The final instrument included 135 representative tasks of HIV management in eleven clusters (Table 1).

**Table 1:** Summary of 135 tasks in HIV care and cadres in Haiti model performing those tasks.

Cluster	Number of tasks	Proportion of tasks in which the cadre participates		
		Doctor	Nurse	Community Health Worker
HIV testing	9	0.89	0.78	0.56
Triage of Patients	1	1.00	1.00	0.00
Clinic Visits	19	0.74	0.79	0.53
Manage stable and complicated patients prior to ART - clinical follow up	27	1.00	0.96	0.56
Manage prior to ART - counseling and education	5	1.00	1.00	1.00
ART Initiation and early follow up (<3 months ART) - counseling and education	2	1.00	1.00	1.00
ART Initiation and early follow up (<3 months ART) - initiation	5	1.00	0.86	0.00
ART Initiation and early follow up (<3 months ART) - clinical follow up	22	0.91	0.91	0.55
Long term follow up of ART after 3 months of ART	19	1.00	1.00	0.55
PMTCT - pregnancy	20	0.90	0.95	0.43
PMTCT - newborn care	6	1.00	1.00	0.83
All tasks (total)	135	0.93	0.92	0.53

ART = antiretroviral therapy.

PMTCT = prevention of maternal to child transmission of HIV.

Between January and June 2007 we conducted a mapping exercise in three clinics in Haiti. The three sites represented different demographics—extremely rural and isolated (Boucan Carré; population 53,000), semi-urban (Lascahobas; population 50,780) and a town (Hinche; population 95,023) all in the Central Plateau department. The Central department is one of the poorest in Haiti, with very limited infrastructure, poor roads, lack of electricity and running water and few secondary schools. It is one of the most isolated departments of the country. Data were collected through semi-quantitative interviews and focus groups with a total of 483 healthcare workers of which 21 were facility-based and 462 were community-based staff. The 21 facility-based staff members were composed of seven different cadres at each of the three sites including the physician and the nurse HIV program managers for the site (n = 6), the chief laboratory technician (n = 3), the chief pharmacist (n = 3), the social worker (n = 3), the midwife (n = 3) and the chief nurse (n = 3). Inclusion criteria and sampling methods for community based health workers at the sites have been described in a previous publication [10].

Staff members providing HIV services were interviewed using the described task-list to quantitatively determine each cadre's areas of responsibility, as well as to elucidate qualitatively their attitudes to and impressions of the model of care. Clinical program outcomes were determined by review of clinic registers and electronic medical records. Quantitative data were compiled using MS Excel and Epi-info6. Qualitative data were recorded and translated from Haitian Creole to English. The goal of the open-ended questions posed during group interviews and focus groups was to search for variability and richness rather than for statistical representation. Analysis was performed by looking for theme and content according to standard qualitative methods [11], [12]. This process included open coding to identify central concepts and categories and axial coding to relate these categories to subcategories [13]. The final coding scheme was developed by consensus of two authors and used for the analysis.

## Results

A large shift in HIV-related tasks was seen in all clinics from doctors to nurses and community health workers compared to a traditional model of care (Tables 1, 2). Only two percent of HIV-related tasks were exclusively performed by clinic doctors, compared to 64% of HIV-related tasks being exclusively performed by doctors in the traditional model of care. Whereas twenty nine percent of HIV-related tasks were traditionally exclusively performed by nurses, only one percent of tasks were exclusive to nurses at Zanmi Lasante. Many tasks at the clinics were shared between doctors, nurses and community health workers. In the traditional model, community health workers had only a small or no formal role in HIV care but in all sites studied, they participated in a large proportion of tasks.

**Table 2:** Tasks exclusively performed by cadre compared to traditional model of care.

Cadre	Traditional	Haiti
Doctor	0.64	0.02
Nurse	0.29	0.01
Non-clinician	0.03	0.04
Doctor/Nurse (shared)	0.01	0.25
Nurse/Community Health Worker (shared)	0.02	0.01
Doctor/Non Clinician (shared)	–	0.01
Doctor/Nurse/Community Health Worker (shared)	–	0.16
Doctor/Nurse/Community Health Worker/Non-Clinician (shared)	–	0.36
Doctor/Nurse/Non-clinician (shared)	–	0.12
Nurse/Community Health Worker/Non-Clinician (shared)	–	0.01
Nurse/Non-Clinician (shared)	–	0.01

Nurses were observed to perform 92% of the 135 HIV-related clinical tasks in the clinics and this included participation in 100% of those related to triage of patients, management prior to antiretroviral therapy (ART), counseling and education around initiation of ART, management of patients on ART after three months of therapy and new-born care tasks related to prevention of maternal to child transmission of HIV (PMTCT). Often nurses initiated management of complicated medical problems, and then consulted a physician for definitive therapy. They prescribed first line ART, but not second-line ART. Of note, among tasks evaluated, nurses did not execute simple laboratory tests or take blood for CD4 testing, nor did they participate in laboratory procedures such as registering results, filling in laboratory result form, performing microscopy or executing X-ray examinations. In terms of clinical care, they did not make decisions for patients with HIV to initiate TB treatment in cases of smear negative or extra pulmonary TB. Furthermore, they did not prescribe or recommend specific ART treatment for patients who were non ART naïve. There were some groups of tasks that nurses did perform that CHW did not participate in at all, including triage of patients at the health center and initiation of ART.

Community health workers made up a large component of staff and ranged from community members who have basic literacy skills and a short three to seven day training course, to those who had completed secondary school and received up to three months of formal classroom and on-the-job training. They participated in over half of all HIV-related tasks, including HIV testing activities, patient visits, management of patients prior to and after initiation of ART, PMTCT and long term follow up of patients on ART. Community health workers were regularly relied upon to provide psychological support, as well as general education on HIV/AIDS. In addition, they facilitated access to clinic-based care, having been trained to identify potential HIV-related illnesses, performed supporting clinical tasks including taking vital signs, administering intramuscular or subcutaneous injections, providing wound care, and monitoring a patient's response to treatment. More complex tasks performed by community health workers prior to initiation of ART, during therapy, as well as in long term follow up included participation in the management of patients with herpes zoster, oral thrush, signs suggesting Kaposi Sarcoma, headache, nausea and fever, primarily by identifying them and facilitating the patients' referral to the health center. They identified patients with chronic symptoms characteristic of co-infection with TB, and dispensed treatment when prescribed by a doctor. Interpretation of laboratory test results and major treatment decisions in addition to supervision of community health workers remained under the purview of doctors and/or nurses. In providing both social and clinical support to patients, community health workers were positioned to recognize and provide assistance to eliminate barriers to adherence. Community members with little formal education also worked in supportive services for HIV including as X-ray technicians, laboratory assistants, pharmacy assistants, and data clerks. The largest cadre of community health workers observed in the model is *accompagnateurs*, described below [9], [14].

Analysis of qualitative interviews indicated that task-shifting was acceptable to staff at all three sites. Doctors reported that they could take on additional subspecialty tasks and would be willing to shift some clinical consultation to nurses. Nurses reported that they could take on both HIV and non-HIV related tasks and identified some that they could shift to lesser skilled workers (e.g. pre and post-test counseling in PMTCT). Nurses and doctors supported the role of community health worker as integral to their own duties and recognized the presence of community health workers as allowing them to spend time on more complicated tasks. Community health workers overwhelmingly took pride in their role in the community and at the health centers. They reported that community members “make us part of their lives”. An *accompagnateur* underlined their role in the social aspects of their patients' lives: “…[with our work] we change the perceptions of the disease…and reduce stigma”. Community health workers also expressed a desire to take on certain tasks that they were not currently assigned to do.

Non-clinician cadres such as pharmacists, social workers, counselors and laboratory technicians all identified tasks that they would be willing to assume. Examples included a social worker who wanted to be able to perform HIV tests directly for clients, especially during home visits. These cadres also identified tasks that they believed could be shifted to lesser skilled staff (including rapid tests for HIV, routine laboratory tests, counseling about ART, stock management, pill counts, and report generation). Universally, willingness to take on new tasks was conditioned upon the need for ongoing training and support, adequate materials and fair remuneration for work performed.

Between October 2002 and October 2007, the network of Zanmi Lasante/Ministry of Health clinics scaled up HIV care to the entire Central Department of Haiti (population 550,000) using the task-shifting model of care, enrolling 11,114 people living with HIV in care, starting 3,763 patients on antiretroviral therapy, with no waiting lists for treatment at the time of the study completion. Twelve month survival of patients ever started on ART in the clinics studied was high at 87.1%, 88.3% and 91.4% with active case-finding [15]. Of those ever starting ART, rates of abandonment were extremely low: 2%, 3.6% and 4.3% at the three clinics respectively.

## Discussion

The magnitude of the HIV epidemic and the severe shortage of physicians and nurses, particularly in resource-poor countries, demand that institutions responsible for healthcare re-evaluate the way that human resources are distributed. The number of individuals requiring HIV treatment and care far surpasses the current capacity of most healthcare systems in the developing world. As part of a comprehensive strategy that includes training new professionals and making efforts to retain existing employees, shifting tasks from doctors to nurses and other cadres of workers has potential to improve efficiency of health systems and to take advantage of human resources that are locally available. Non-physician clinicians have been described in many countries in sub-Saharan Africa, as well as in high-income settings [3], [16], [17] but few data exist on the effectiveness of task-shifting as a model of care for scaling-up access to HIV services, HIV program outcomes, or acceptability to staff and patients [18]. Haiti faces a serious gap in healthcare workers. The public sector has just 0.08 doctors per 1,000 population, nursing ratios are 0.12 per 1,000 population [6], [19]. Even this is an underestimate of the problem, as skilled health workers tend to be located mostly in the cities and not in rural areas such as those served by the clinics studied. UNAIDS estimated the number of people living with HIV in Haiti to be 190,000 in 2006 [20].

This case study demonstrates that task-shifting to nurses and community health workers is effective in terms of scaling up care and safe in terms of program outcomes. There were extremely low rates of abandonment at all three sites studied—retention in care was far superior to that reported in a meta-analysis of loss to follow up in sub-Saharan Africa, in which the best retention rate was 90% at 24 months and the average retention rate was 60% at 24 months [21]. Since adherence to ART is known to be essential for good outcomes [22], retention of patients in care is a critical first step to both ensuring their own health and in the prevention of development of resistance to ART. Twelve month survival on ART at the sites studied was comparable to published data from similar resource-poor settings [23], [24]. Although it is not proposed that the results of the program in terms of retention in care and mortality are outcomes of the human resources model alone, they provide context that justifies ongoing interest in the model as part of a successful comprehensive strategy for HIV care.

This case study highlights the innovative use of CHW in HIV care, detailing their HIV-related tasks and, in particular the sharing of tasks between CHW and nurses, as well as doctors at the sites. The most widely employed CHW in the Haiti model studied are *accompagnateurs*—health workers that deliver medications to the homes of those on ART and observe doses being taken [9]. They also assist in active follow up by locating patients who may have moved or migrated in search of economic needs. In this role, CHW performed certain tasks that were largely not performed at all in a traditional doctor-centered model of care, specifically by acting as the ‘bridge’ to health services from the community. By recognizing symptoms of opportunistic infections and side effects of medications *accompagnateurs* act as an essential component of the clinical team.

Staff members at all levels were accepting of the task-shifting model, but all cadres reinforced the need for ongoing training, supervision as well as adequate salaries for activities and the need for steady stocks of materials and supplies. Importantly, task-shifting did not decrease the need for more senior cadres—roles of senior staff changed rather than decreased and doctors and nurses were required for supervision, training and mentorship as well as for care of complicated patients. Failures of community health workers programs have been reported in other settings; however, analysis of these failures often demonstrates that they were due to a lack of adequate support, training, remuneration or supervision [25]–[29]. Further analysis of the impact of CHW in this Zanmi Lasante/Ministry of Health model on the health system as a whole in rural Haiti is described elsewhere [10].

Though not consistently termed task-shifting, the practice is not new in either high-income countries or many middle and low-income countries [16], [30], [31]. Previous studies have demonstrated the success of involving less skilled cadres of workers in health care programs from malaria control to cesarean sections [32]–[36]. In our program in Haiti, the practice of task-shifting was originally focused on tuberculosis treatment and expanded to encompass HIV/AIDS treatment programs. Subsequently, that model of care was exported to programs in Boston, Rwanda and recently Lesotho and Malawi. In an environment where highly skilled health workers were almost non-existent, expansion of the role of less skilled individuals was essential [14], [22], [37]. Of note, most tasks were shared between cadres rather than the role of one cadre being replaced by another. It may be more appropriate to call this approach ‘task-sharing’, as others have also suggested [38].

The case study is limited by being an observational study. Given the health worker crisis and the realities of the field, it was difficult to find geographically comparable models of care in Haiti where task-shifting was not taking place to any extent. A second limitation is the use of recall to determine participation in HIV-related tasks as these may be subject to bias, this is also true in relation to the development of the baseline task list. Furthermore, it is important to note that reorganization of healthcare teams is not alone sufficient for success in the model of care—it is comprehensive, using a primary care approach to the prevention, diagnosis and treatment of HIV, has a solid procurement system, collaboration with the Haitian ministry of health and attention to the socioeconomic needs of patients. Despite these other factors, however, because of lack of doctors and nurses, as well as the rural, isolated nature of the terrain, scale up of HIV care and primary care services in central Haiti could not have successfully happened if task-shifting had not occurred.

### Conclusion

Task-shifting exists as a successful model for scale up of HIV care in central Haiti, with good clinical outcomes and excellent retention in care. Where human resources for health are limited, task-shifting from doctors to nurses and community health workers may be undertaken as a strategy for rationally distributing human resources when certain conditions are met. Shifting tasks to community health workers provides an additive benefit regardless of the healthcare worker crisis; that is they provide certain essential services such as adherence support and psychosocial counseling that are not easily delivered by clinic-based staff alone.
